# Hepatic Cysts in the Left Triangular Ligament: A Rare Finding with Potential Diagnostic Challenges

**DOI:** 10.5334/jbsr.3952

**Published:** 2025-04-16

**Authors:** Jan Lievens, Bart Lutin, Frédéric Vanhove

**Affiliations:** 1 AZ Groeninge, Kortrijk, Belgium

**Keywords:** Hepatic cyst, left triangular ligament, left upper quadrant lesion, gastric mass mimic, abdominal mass

## Main Teaching Point

Hepatic lesions, originating from the left triangular ligament (LTL)—though rare but potentially malignant (e.g. hepatocellular carcinoma) or often mimicking gastric masses—should be included in the differential diagnosis of left upper quadrant lesions to prevent diagnostic delays and unnecessary interventions.

## Case History

**Figure 1:**
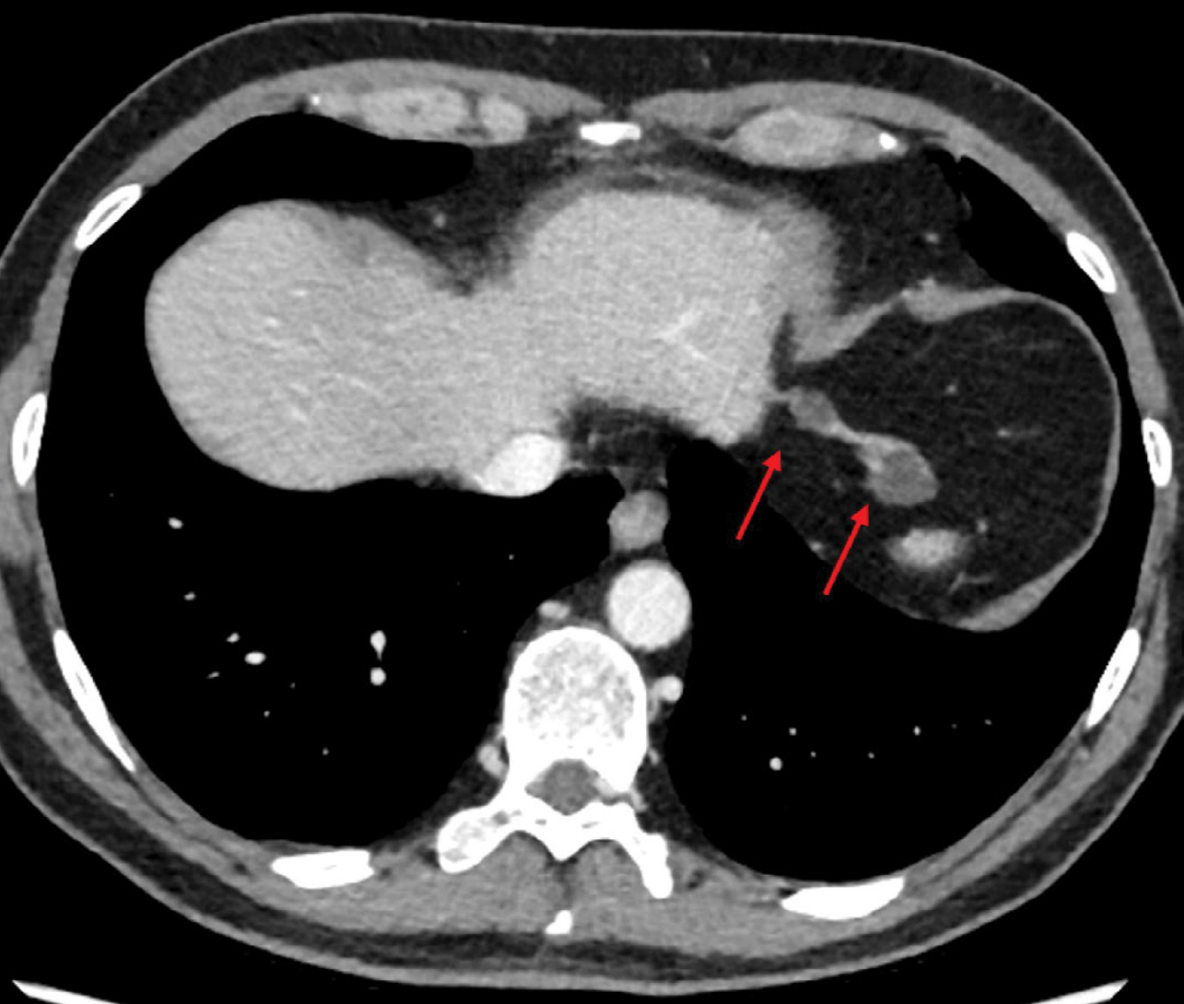
Axial view of the contrast enhanced abdominal CT‑scan showing the cystic lesions (arrows).

**Figure 2:**
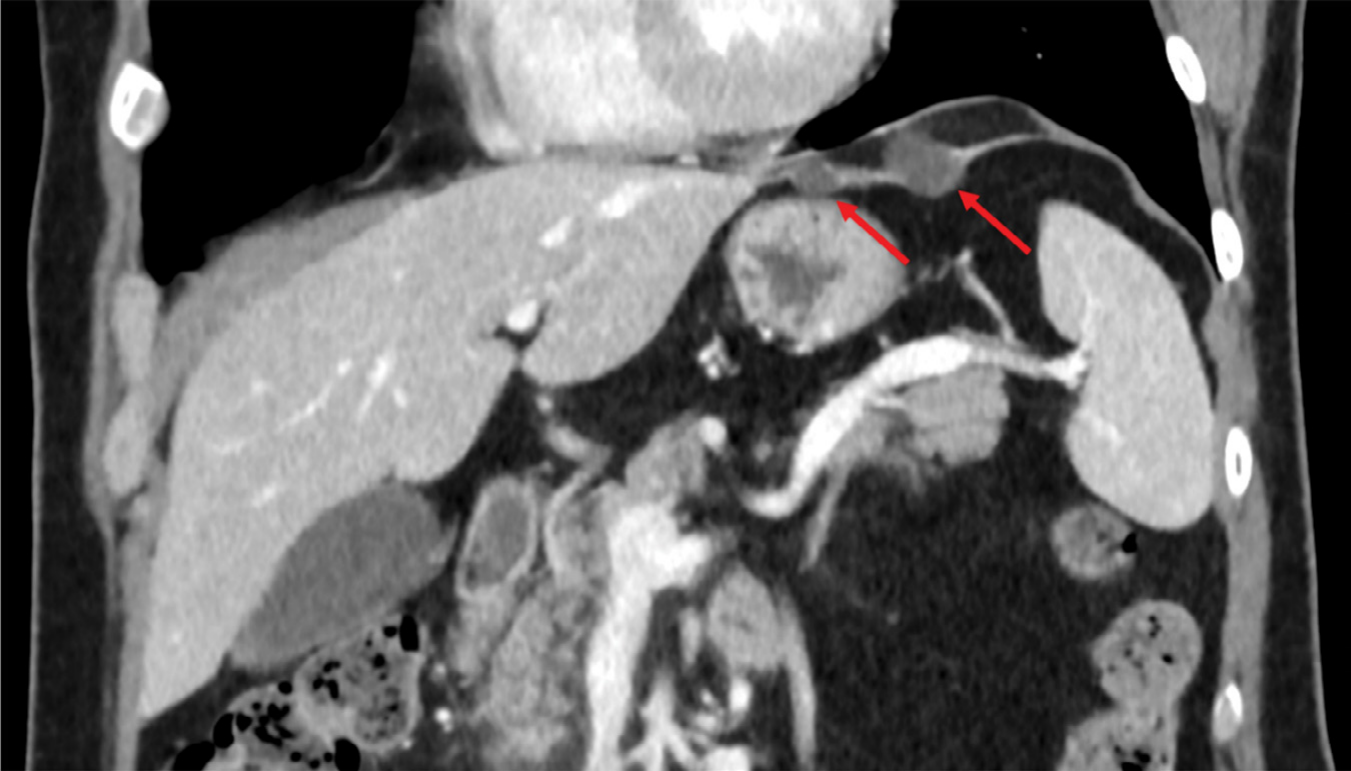
Corional view of the contrast enhanced abdominal CT‑scan showing the cystic lesions (arrows).

A 55‐year‐old male was referred for a contrast‐enhanced abdominal computed tomography (CT) scan due to persistent abdominal pain. CT revealed two subdiaphragmatic thin‐walled hypodense cystic lesions (3 HU) in the left hypochondrium (largest diameter 22 mm and 12 mm). A thin strand of tissue connected the lesions to each other, to the liver, and to the diaphragm (Figures 1 and 2). These lesions originate from the left hepatic triangular ligament and are regarded as hepatic cysts.

## Comments

**Figure 3:**
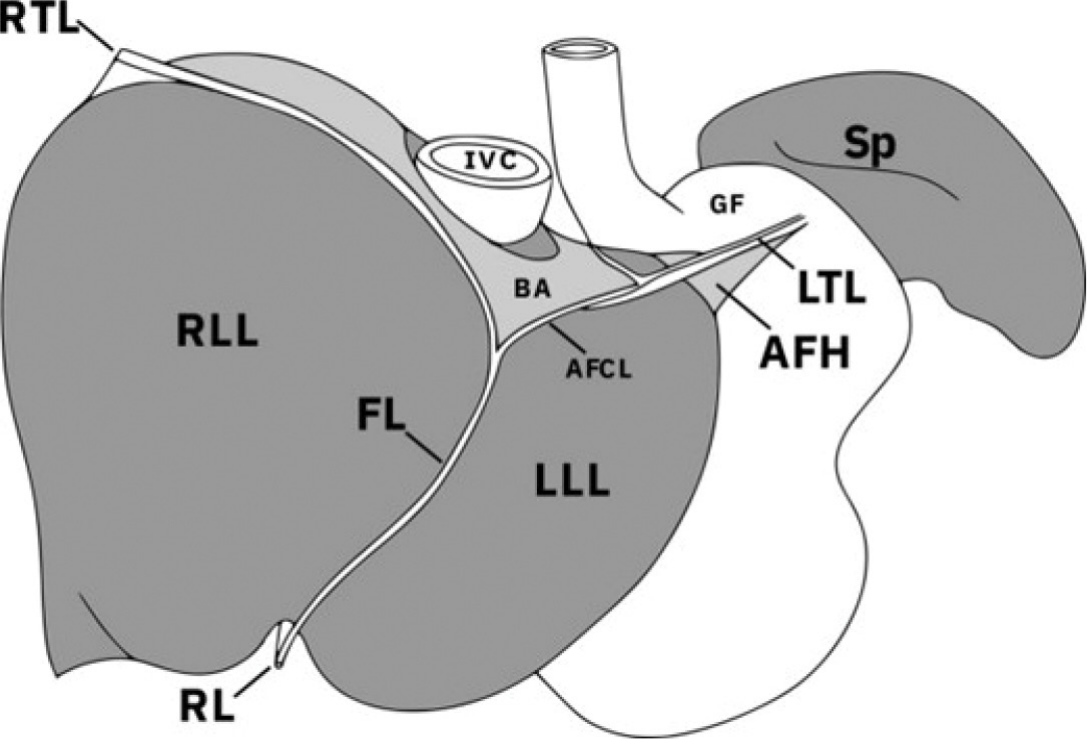
Ligaments of the liver and their relation to the other upper abdominal viscera adjacent to the diaphragm. LTL left triangular ligament, AFH appendix fibrosa hepatis, AFCL anterior fold of the coronary ligament, FL falciform ligament, RL round ligament, RTL right triangular ligament, RLL right lobe of liver, LLL left lobe of liver, BA bare area, IVC inferior vena cava, GF gastric fundus, Sp spleen [1].

The left triangular ligament is a peritoneal fold that anchors the left lobe of the liver to the diaphragm. It is formed by the convergence of the coronary ligament on the left side and serves as the counterpart to the right triangular ligament (Figure 3).

Cadaveric studies have shown that LTLs often contain hepatic tissue and, consequently, may harbor hepatic cysts [1]. However, (iatrogenic) bile leaks, hemangioma, and malignant lesions such as hepatocellular carcinoma have also been reported in this location. Given the potential for malignancy, any mass in the subdiaphragmatic area of the left upper quadrant (LUQ) should raise suspicion of an LTL origin to minimize diagnostic delays and prevent unnecessary procedures.

The diagnostic challenge frequently lies in distinguishing a LUQ mass of gastric origin from a mass arising in the LTL. Special attention is required when a mass appears to arise from the stomach near the gastric fundus with an exophytic growth pattern or if a linear structure (the LTL) is observed connected to the left hepatic lobe or diaphragm. If the stomach wall can be delineated separately from the mass, an alternative origin should be considered. Characterization of the origin becomes more challenging when the LTL is not clearly visible [1]. Nevertheless, visibility of the LTL’s may be challenging owing to the wide range of variability in length and position of the LTL or owing to respiratory motion artifacts.

In summary, lesions arising from the left triangular ligament are rare and typically of hepatic origin, including potentially malign (pseudo‑)lesions. Occasionally, LTL‑originating lesions may be difficult to distinguish from gastric masses and therefore should be included in the differential diagnosis of LUQ lesions—especially when the LTL is not clearly delineated or the mass exhibits a predominately exophytic growth from the stomach, often confirmed by gastroscopy. The radiologist’s attention should be drawn to this anatomic variant to avoid diagnostic delays and unnecessary interventions.

## References

[r1] Agarwal S, Munyal R, Aravinthan A, Clarke C. Left triangular ligament lesions are likely hepatic in origin: A systematic review. Br J Radiol. 2023;96(1152):20230231. DOI: 10.1259/bjr.20230231. 37747273 PMC10646653

